# Toward Understanding the Molecular Bases of Stretch Activation: A STRUCTURAL COMPARISON OF THE TWO TROPONIN C ISOFORMS OF LETHOCERUS*

**DOI:** 10.1074/jbc.M116.726646

**Published:** 2016-05-20

**Authors:** Domenico Sanfelice, Máximo Sanz-Hernández, Alfonso de Simone, Belinda Bullard, Annalisa Pastore

**Affiliations:** From the ‡ Department of Clinical and Basic Neurosciences, Wohl Institute, King's College, London SE5 3RT, United Kingdom,; the § Department of Life Sciences, Imperial College, London SW7 2AZ, United Kingdom,; the ¶ Department of Biology, University of York, York YO10 5DD, United Kingdom, and; the ‖ Department of Molecular Medicine, Universita' of Pavia, Pavia I27100, Italy

**Keywords:** muscle, protein-protein interaction, stress, structural biology, structure-function

## Abstract

Muscles are usually activated by calcium binding to the calcium sensory protein troponin-C, which is one of the three components of the troponin complex. However, in cardiac and insect flight muscle activation is also produced by mechanical stress. Little is known about the molecular bases of this calcium-independent activation. In *Lethocerus,* a giant water bug often used as a model system because of its large muscle fibers, there are two troponin-C isoforms, called F1 and F2, that have distinct roles in activating the muscle. It has been suggested that this can be explained either by differences in structural features or by differences in the interactions with other proteins. Here we have compared the structural and dynamic properties of the two proteins and shown how they differ. We have also mapped the interactions of the F2 isoform with peptides spanning the sequence of its natural partner, troponin-I. Our data have allowed us to build a model of the troponin complex and may eventually help in understanding the specialized function of the F1 and F2 isoforms and the molecular mechanism of stretch activation.

## Introduction

Mechanical stress is an important element in cellular processes as different as muscle contraction, external mechanical loading, cell migration, and protein aggregation and misfolding. Among these processes, muscle contraction involves considerable stress that results from the relative movement of parallel thick and thin filaments (1), respectively formed predominantly of myosin and actin. Cyclic interaction of the motor domain of myosin with actin propels the sliding filaments. In striated muscle, the interaction of myosin with actin is controlled by tropomyosin (Tm)^2^ and troponin (Tn), which are periodically arranged along the thin filaments (2, 3). The Tn complex has three subunits: TnT binds to Tm, TnI binds to actin and holds Tm in a blocking position, and TnC binds Ca^2+^ and detaches TnI from actin, allowing Tm to move. TnI and TnT are intrinsically unfolded in isolation but become mostly helical upon ternary complex formation (4, 5). TnC has a typical dumbbell structure with two globular domains, each of which contains two EF-hand motifs (6). In relaxed muscle, the interaction between myosin and actin is blocked by Tm (3, 7–9). Ca^2+^ influx triggers a conformational transition of Tn, which releases the inhibition by displacing Tm and exposing the myosin-binding sites according to the so called steric blocking model (3). In addition to Ca^2+^ activation, all striated muscles are also activated by mechanical stress. When an active muscle is stretched, it produces more force after a short delay (10, 11). This additional activation is particularly prominent in cardiac muscle and in the indirect flight muscle (IFM) of insects (12, 13). The mechanism by which fibers sense stretch is not known, despite its importance for a basic understanding of muscle contraction and for its implications for myopathies. Insect flight muscle is an extreme example showing stretch activation, making IFM an ideal model system. Insect wings are moved by opposing pairs of muscles acting alternately, with one muscle contracting and shortening while the other is stretched (14–16). Previous work has shown that stretch activation in IFM works by the steric blocking mechanism in which Tn plays an important role (17, 18). TnC is present in IFM in two isoforms. The major one (90%), F1TnC, binds a single Ca^2+^ ion in EF-hand 4 and is needed for the response of IFM to stretch (14, 19). The minor isoform, F2TnC, is only 10% of the TnC and binds two Ca^2+^ ions in EF-hands 2 and 4 (14, 19). Mechanical measurements with *Lethocerus* IFM fibers in which TnC isoforms were exchanged suggest that F1TnC is important for stretch activation, whereas F2TnC is required for calcium-activated isometric tension (14, 20). This difference in properties was suggested to be due to structural differences or to formation of different interactions with other proteins (20). The structure of F1TnC was solved. The most noticeable difference from TnC in vertebrate muscle is that binding of only one Ca^2+^ in the fourth EF-hand motif is sufficient to induce a closed to open conformational transition in the C-terminal lobe (19, 21). Much less is known about F2TnC, making it difficult to test these hypotheses.

To fill the gap and gain further understanding of stretch activation, we focused here on F2TnC. Using an NMR study, we compared structural and dynamic properties of this protein with those of F1TnC. We also map the interactions of F2TnC with TnH (the *Lethocerus* equivalent of TnI) using synthetic peptides and the reconstructed ternary complex. Our data show that, although the two isoforms have a similar structure, they have distinct dynamic behavior in solution, which may have profound consequences on how the two isoforms function and on their mechanical properties.

## Results

### 

#### 

##### Comparison between the Calcium-loaded and Calcium-free Forms of F2TnC

**FIGURE 1.**
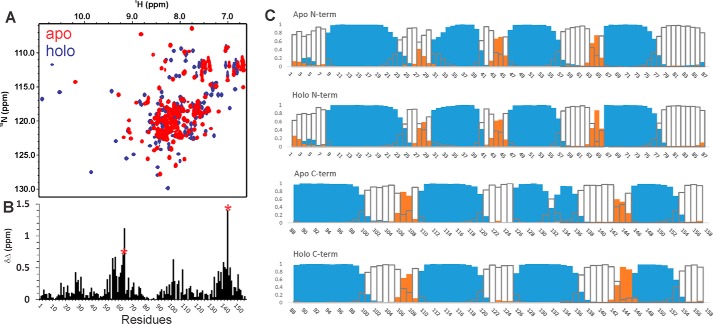
**Differences between the spectra of apo and holo F2TnC.** *A*, spectrum superposition of the two proteins with the holo form in *blue* and the apo one in *red. B*, plot of the secondary structure along the sequence as suggested by the Talos program, calculated on backbone chemical shifts. *Cyan columns* indicate helical regions, *orange columns* β sheet regions, and *empty columns* random coil conformations. *C*, chemical shift perturbation upon calcium binding. Leu-65 and Val-144, which are in position 8 of the respective calcium binding loops according to the canonical EF-hand loop numbering, are indicated with *red stars*. Residues Glu-66 and Val-144 present the highest values.

The NMR ^1^H,^15^N HSQC spectra of apo and holo F2TnC both have good spectral dispersions, indicating that they are intrinsically structured also in the absence of Ca^2+^ (Fig. 1*A*). This is in contrast to the behavior of the C lobe of skeletal muscle TnC, which is unstructured in the absence of Ca^2+^ and has thus a spectrum with low chemical shift dispersion (22). The secondary structure content is consistent with these observations. As evidenced by plotting the secondary structure calculated by the Talos program (23), it is clear that both apo and holo forms are helical with a content that is comparable (Fig. 1*B*). The role of calcium is thus not structural because F2TnC is folded also in the absence of the cation.

Calcium induces clear chemical shifts only in some resonances around EF-hands 2 and 4, which is where calcium binds (6) (Fig. 1*C*). The two other coordination sites are inactive and thus expected to be unaffected by Ca^2+^. The chemical shift variations between the apo and holo forms have an interesting peculiarity. Only one resonance, which corresponds to Val-144, shifts significantly and moves from the center of the spectrum of the apo to 9.9 and 127.5 ppm in the holo form. This behavior is typical for the amide of the hydrophobic residue in position 8 of the calcium binding loop according to the canonical EF-hand loop numbering and is highly diagnostic of the presence of Ca^2+^ binding (6, 24). A smaller shift (0.6 *versus* 1.4 ppm in terms of Δδ) is observed for the resonance of the corresponding position in the N lobe of EF-hand 2 (Leu-65), which contains the second calcium binding site. This is probably due to the lower affinity of this site compared with that of EF-hand 4 (93 *versus* 6 μm) (25). Residue Glu-66 shows an unusual chemical shift variation (1.1 ppm) that suggests a slightly different conformation of the loop and/or a distorted Ca^2+^ coordination.

##### Comparison of the Spectra of Full-length F2TnC and Its Fragments

**FIGURE 2.**
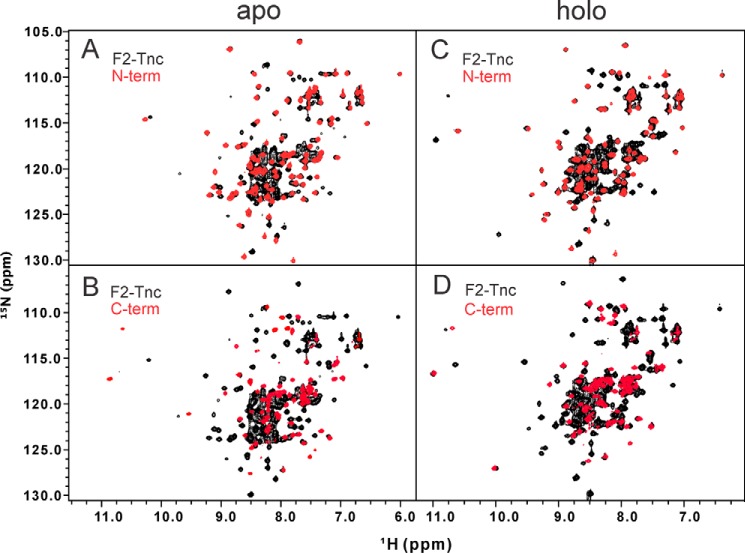
**Assessing interactions between lobes.** Comparison of the spectra of the isolated apo (*A* and *B*) and holo (*C* and *D*) forms of the N and C lobes with full-length F2TnC. In both images, full-length F2TnC is shown in *black* and the N lobe and the C lobe in *red*. The HSQCs of the holo fragments almost completely overlap on the full-length construct, indicating the absence of interaction between lobes. This is not completely true for the apo forms.

To obtain further information on the protein, we produced separately the isolated N and C lobes (residues 1–88 and 89–158, respectively). Comparison of the spectra of the individual fragments shows a behavior consistent with what is observed with the full-length protein (Fig. 2). The spectra of the two lobes are well dispersed. This means that the two regions fold independently, as also observed in F1TnC (19). An interesting feature, however, is that the spectrum of the isolated C lobe is as intense as that of the isolated N lobe, whereas, in full-length F2TnC, the resonances of the C-terminal region are less intense than the N-terminal ones. This suggests different dynamic features of the two regions and the presence of a differential conformational exchange.

Superposition of the spectra of full-length F2TnC with its fragments indicates some rearrangements in the apo forms, whereas the spectra of the holo forms are almost completely additive, indicating lack of interactions between the two lobes. Additivity of the spectra of the N- and C-terminal halves of the protein is a feature common to calmodulin and most EF-hand proteins (26).

##### Comparisons between F1 and F2TnC Spectra

**FIGURE 3.**
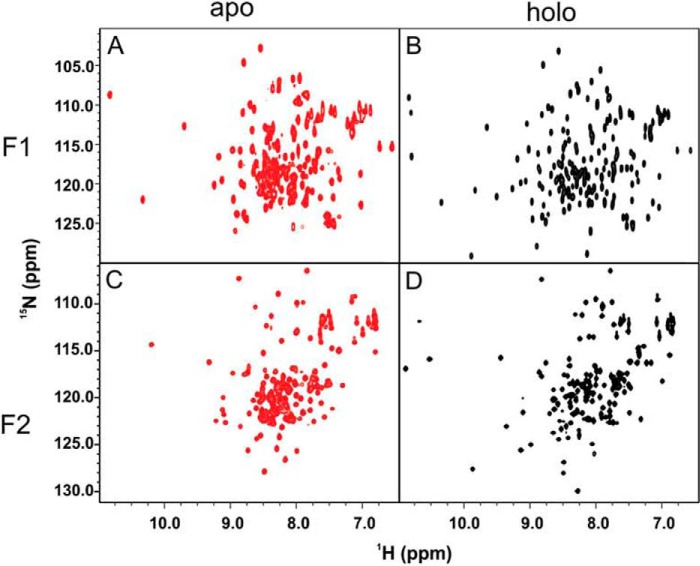
**Comparison between F1 and F2TnC.** ^15^N HSQC spectra of the two apo and holo forms of F1 (*A* and *B*) and F2 TnC (*C* and *D*). The figure highlights the differences in calcium recognition of the two isoforms.

The HSQC spectra of full-length F1TnC and F2TnC show expected differences (Fig. 3) given that the two sequences share 47% identity. The spectrum of apo F2TnC has a relatively good dispersion that is overall comparable with that of apo F1TnC, suggesting a similar level of retained tertiary structure. When calcium is present, both spectra become more dispersed, but F2TnC remains slightly more collapsed. This is unexpected given the presence of two Ca^2+^ binding sites in F2TnC compared with only one in F1TnC. Comparison between the full length and the two fragments (Figs. 2 and 3) tells us, however, that the C lobe has an excellent dispersion in the holo but not in the apo form. This strongly suggests that this lobe undergoes an appreciable closed-to-open transition, as is the case for F1TnC. More difficult is to predict at this stage whether the N lobe also changes upon calcium binding, and this will have to be assessed more carefully in the future. However, the relatively small chemical shift changes observed upon calcium addition and comparison with the spectra of apo and holo F1TnC (Figs. 1 and 3) suggest that, if at all, the conformational change will be minimal.

##### F1TnC and F2TnC Have Similar Dynamic Properties

**FIGURE 4.**
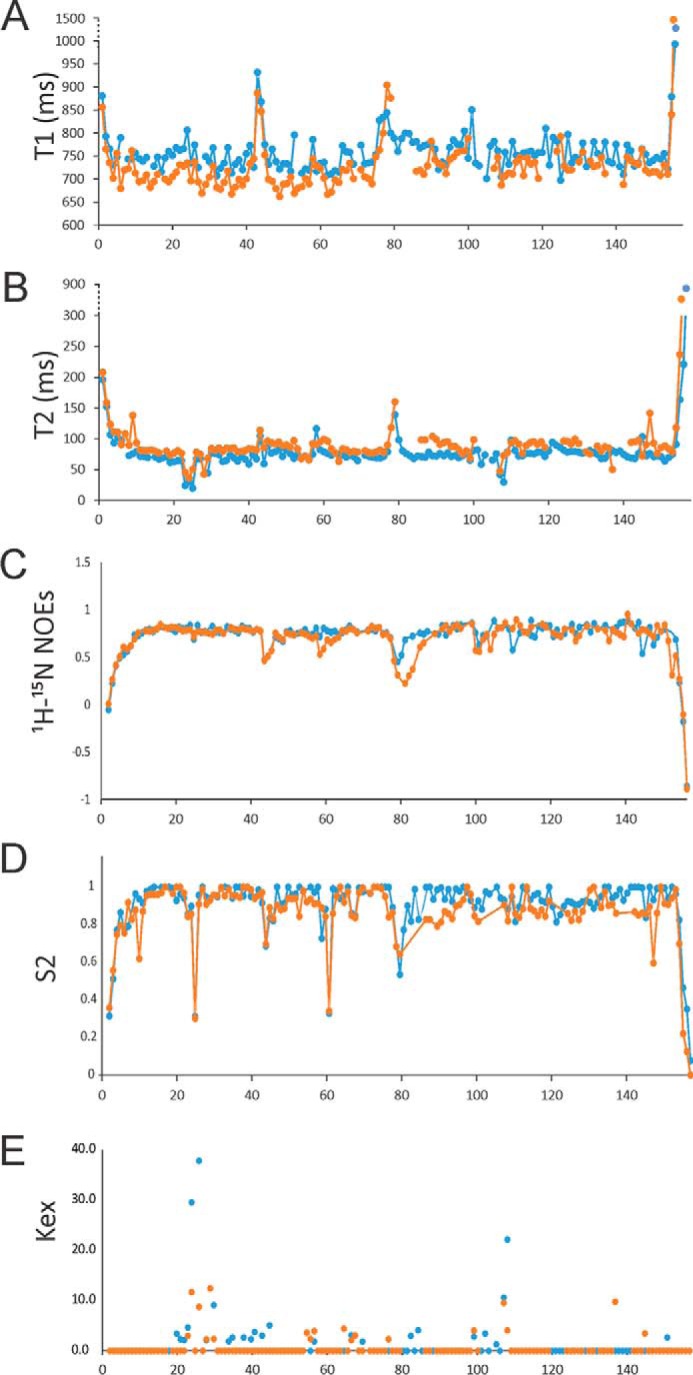
**Dynamic properties of F2TnC.** Shown are T_1_ (*A*), T_2_ (*B*), heteronuclear ^1^H-^15^N NOE (*C*), S^2^ (*D*), and exchange term (*Kex*, *E*) values. The apo and holo forms are indicated in *blue* and *orange*, respectively.

We then compared the dynamic properties of F2TnC with those of the F1 isoform. T_1_, T_2_, and NOE of full-length F2TnC (Fig. 4) are overall similar to those described previously for the corresponding region of full-length holo F1TnC (19). The average T_1_ value in the apo form calculated from residues with NOEs of >0.6 (that is, residues whose relaxation is not significantly affected by internal motions) is 475.7 ± 24.0 ms; the equivalent value for the holo form is 479.8 ± 20 ms. Small deviations are observed in the loop regions, in agreement with the root mean square deviation data. Noticeable differences are instead observed for the transversal relaxation T_2_ values, which are overall significantly smaller for the apo form, especially for residues in the loop regions. The average T_2_ values are 115.1 ± 34.9 ms and 155.3 ± 7.25 ms for the apo and holo forms, respectively.

Most of the data could be fit satisfactorily with the simplest model (model 1). Exchange terms had to be used to fit a few residues that typically are in loops 1 and 3 of the EF-hands. This is consistent with these loops not being able to bind to calcium. The loop connecting the two lobes is also overall more flexible. We estimated a correlation time (τ_c_) of 8.4 ns for the full-length apo protein, whereas that of the holo form is slightly longer (9.4 ns). The τ_c_ of the isolated apo N and C lobes are 4.7 and 5.8 ns, respectively. The corresponding values for the holo forms are 4.2 and 5.6 ns, respectively. These values are comparable with the values found for F1TnC (19). These data indicate that, independently of the presence of calcium, F2TnC is formed by two compact and relatively rigid globular lobes.

##### Interaction of F2TnC with TnH/TnI

Having complete assignment of the spectra of F2TnC, we could now map the regions of the protein affected by titration of ^15^N-labeled TnC with two insect TnH/TnI peptides, TnH(30–61) and TnH(126–159). They were designed by homology with regions affected in the interaction in vertebrates (19). TnH(30–61) binds to F2TnC with a *K_d_* of 8 nm in the presence of Ca^2+^, whereas *K_d_* values of 40 nm were found in the absence of Ca^2+^ or in the presence of the more weakly coordinated Mg^2+^ (25). For comparison, F1TnC binds the same peptide with *K_d_* values of 2 and 0.9 nm in the absence and presence of Ca^2+^, respectively. The affinities of TnH(126–159) for F1TnC and F2TnC are almost the same and around 13 and 3 μm in the absence and presence of Ca^2+^, respectively.

We carried out the titrations both with full-length holo F2TnC and with the isolated holo N and C lobes because the fragments have intrinsically more resolved spectra. The results show qualitatively the same behavior, confirming that the N and C lobes work independently.

**FIGURE 5.**
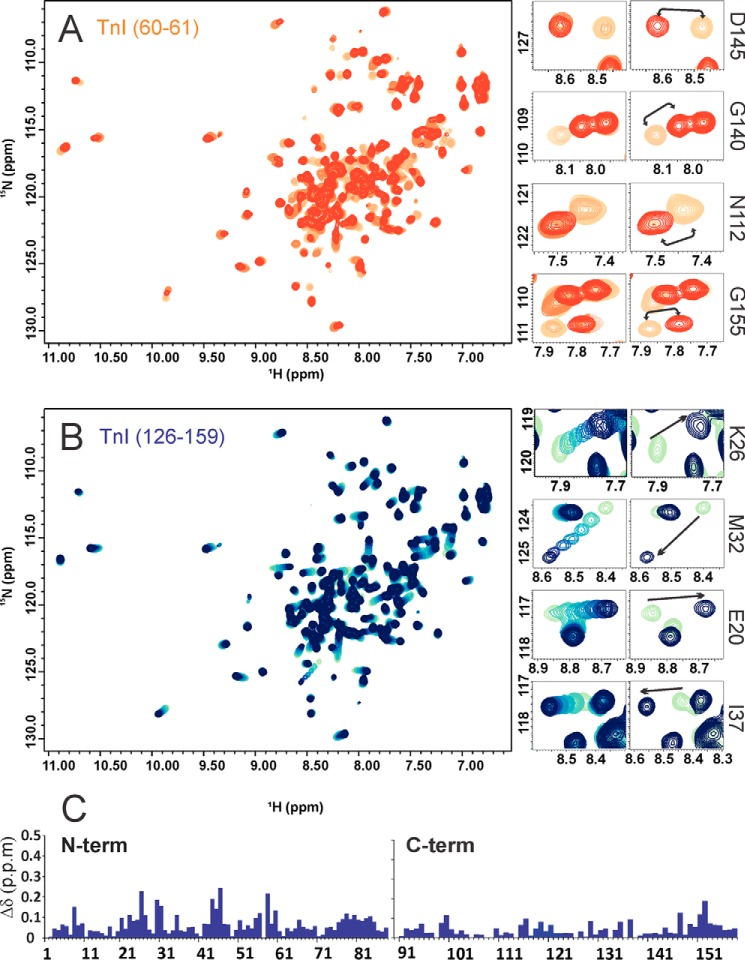
**Testing the interactions of TnH/TnI with F1TnC.** Shown are the effects of the TnH peptide titrations on F2TnC. *A*, *left panel*, spectrum of full-length holo F2TnC titrated with TnH(30–61). *Center small panels* (*All*), close-ups of specific peaks, all belonging to the C lobe, followed throughout the titration. The spectra are progressively colored from *orange* (the starting point) to *red* (final point). The spectra clearly show that the peaks do not move during the titration. Close-ups (*right panels*, *1:3*) of the starting (*orange*) and the final points for the same peaks shown before to highlight the overall displacement (*black arrows*). *B*, spectrum of holo full-length F2TnC titrated with TnH(126–159) (*left panel*). Close-ups of specific peaks, all belonging to the N lobe, followed throughout the titration (*center small panels*). They are color-coded using progressive shades of *blue* from the first (*light green*) to the last (*dark blue*) titration point, clearly showing a progressive chemical shift. The same peaks are shown at the beginning and final points of the titration (*small right panels*, *1:3*). *C*, histogram of the chemical shift perturbation caused by TnH(126–159) on the two F2TnC fragments. It is clear that most of the shifts affect the N lobe. The spectra were all recorded at 25 °C and 600 MHz.

The two peptides affect the spectra in a very different way. Upon titration of F2TnC with TnH(31–60), several resonances do not shift but double, indicating the co-detection of the peptide-bound and unbound forms of the proteins (Fig. 5*A*). This happens already at molar ratios of TnC(31–60):F2TnC below 1:1 and means that these resonances are in a slow exchange regime in the NMR time scale as expected for a tight binding. The affected resonances mostly belong to the C lobe. The unbound form progressively disappears, leaving only the bound form. Some changes are also visible in peaks belonging to the N lobe, but they are in a fast exchange regime of binding because they experience chemical shift perturbation. This tells us that TnH(30–61) binds to both lobes but with different affinities. Binding occurs preferentially on the C lobe, but the N lobe is also able to bind.

TnH(126–159) preferentially binds the N lobe in a fast exchange regime (Fig. 5, *B* and *C*). Some minor perturbation of the C lobe is observed but only at high molar ratios (5:1). The behavior of F2TnC is thus distinct both from F1 and vertebrate TnCs (27, 28) but overall more similar to the cardiac and skeletal proteins than to the F1isoform.

##### Modeling the Lethocerus Tn Heterotrimer

**FIGURE 6.**
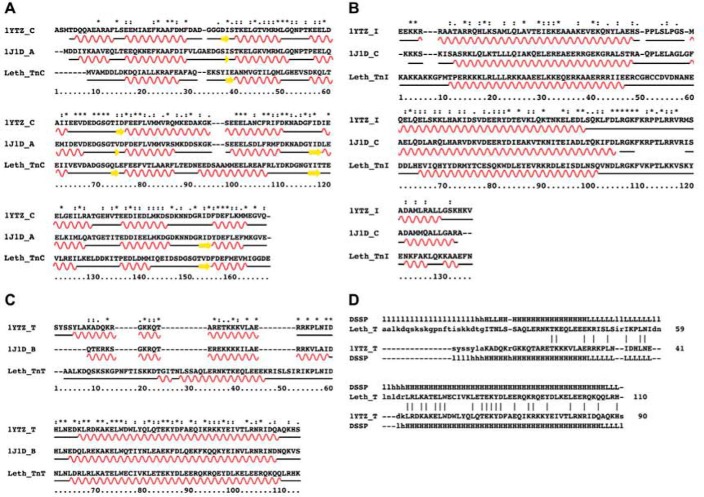
**Modeling the structure of the Tn complex.** *A–C*, sequence alignments used in modeling the three TnC, TnH/TnI, and TnT chains. For TnT, the sequence alignment results in three major insertions (*C*). *D*, structural alignment obtained on the model resulting from our iterative procedure. The helical regions are now aligned, and the gaps are grouped in the loop between the helices and at the N terminus. For each alignment, the template sequences of the human complex (1YTZ and 1J1D) are shown at the *top*, with the *Lethocerus* sequence at the *bottom*.

**FIGURE 7.**
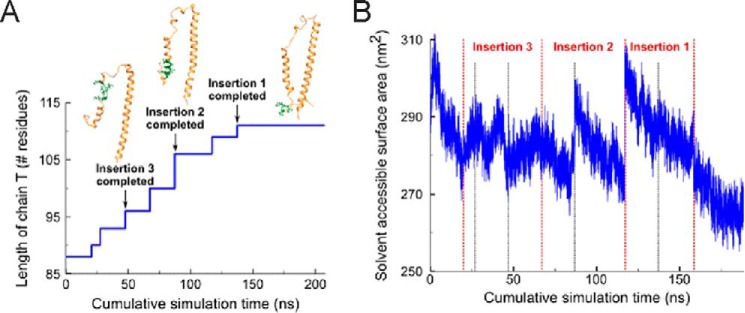
**Gap growing process of *Lethocerus* TnT.** *A*, residues were introduced iteratively at the insertions, allowing the system to relax after each addition. *B*, evolution of the solvent-accessible surface area of the Tn trimer during the gap growing process. *Discontinuous lines* represent the addition of new residues. Growing the chain generally causes abrupt increases in the solvent-accessible surface area, which decreases as the system relaxes to adopt a more globular structure during the MD runs. After completing every major insertion, the system was simulated until the solvent-accessible surface area decreased back to the initial reference value (pre-insertions), indicating that the overall globular fold of the complex has been conserved.

Reassured by our analysis, we modeled the structure of the *Lethocerus* F2-Tn complex using the coordinates of both the vertebrate skeletal and cardiac complexes as templates (PDB codes 1YTZ and 1J1D). These are currently the only two high-resolution structures available for the Tn trimer, probably because the intrinsic flexibility of all the components makes this complex unsuited for crystallization. The two structures greatly differ for the orientation of TnC relative to the TnI and TnT chains. We first considered calcium-loaded skeletal muscle 1YTZ. Compared with the sequences of this complex, *Lethocerus* TnC and TnH/TnI do not have major insertions or deletions (Fig. 6, *A–C*). Standard homology modeling could thus be used to build their structures. In contrast, *Lethocerus* TnT presents three insertions that are too large to be modeled with standard loop modeling methods (29). Additionally, one of the insertions is in the middle of the first helix of the TnT hairpin, which poses the question of whether this region should also be helical. To circumvent this problem, we modeled the insertions by an iterative gap growing process, whereby residues were gradually added to fill in the gaps. After each insertion was completed, the system was sampled through short molecular dynamics runs until the overall fold had sufficiently relaxed toward that of the native homology template (Fig. 7). In this way, we could progressively lengthen the peptide chain to its full length while maintaining the structure of the trimer in an energetically favorable state, which remains conformationally similar to the homologous template. After the model was complete, an MD simulation was calculated with these coordinates for an additional 20 ns under a simulated annealing protocol to generate an ensemble of structures. The potential energy of the ensemble was estimated according to the force field employed in the simulations, giving a value of −7682 ± 207 kcal/mol. We generated a second model using the cardiac muscle calcium-loaded 1J1D template with alternative orientation of the C chain and combined it with the previously modeled TnH/TnI and TnT. Also, the second heterotrimer was submitted to 20-ns dynamics with simulated annealing to generate a second ensemble of structures. The potential energy of this ensemble is −8134 ± 217 kcal/mol.

**FIGURE 8.**
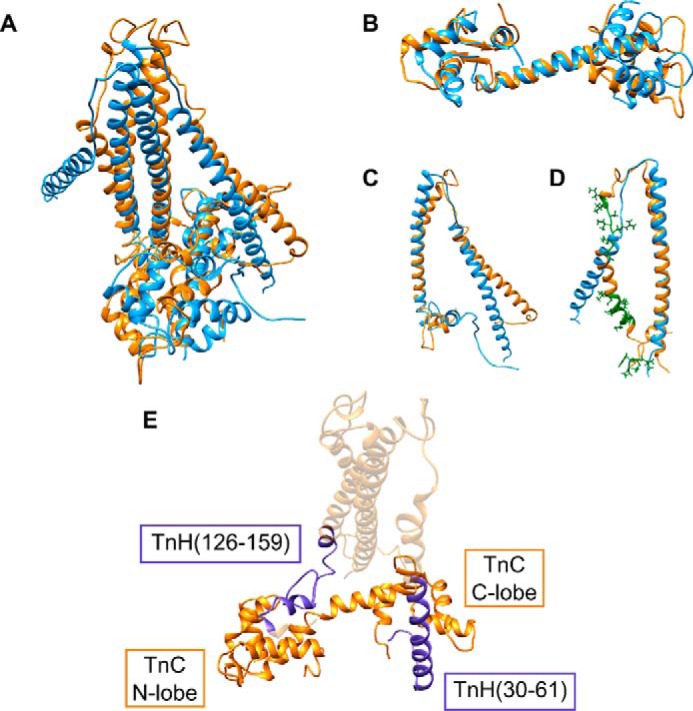
**Structural comparison between the *Lethocerus* Tn complex and vertebrate x-ray structures.** *A*, structural superposition of the template structure of the skeletal Tn trimer 1YTZ (*blue*) and the *Lethocerus* model (*orange*). *B–D*, superposition of the individual chains TnC (*B*), TnH/TnI (*C*), and TnT (*D*). Large insertions modeled by iterative gap growing are shown in *green. E*, mapping of the TnH peptides (*purple*) onto the trimer structure, showing their interactions with TnC.

Structural superposition of the resulting TnT model on the template provides a good fit when we shift the alignment of the skeletal TnT forward along the sequence of *Lethocerus*. This produces a unique insertion in the *Lethocerus* sequence in the hairpin loop (Fig. 6*D*). The overall shape of the complex remains similar to the templates, but the orientation of the N-terminal helix of TnT slightly differs (Fig. 8). The pattern of intermolecular interactions between TnH/TnI and TnC is unaltered with respect to the template, and the structure remains stable throughout the MD sampling. Specifically, the mapping of the TnH peptides onto the structure of the modeled complex is in excellent agreement with our titration data (Fig. 8*E*). These models thus recapitulate all the important features of the complex and provide an excellent starting point for further studies that might allow their validation.

## Discussion

Both cardiac and insect flight muscles are activated by stretching. However, little is known about the molecular basis of either length-dependent or stretch activation, although they are involved in cardiomyopathies. Several mechanisms have been suggested to explain it (17, 30–36), but none have been proven. The identification of two isoforms of TnC in IFM, F1TnC, which is necessary for stretch activation and oscillatory contraction, and F2TnC for development of isometric force, suggested that a study of the structure of the isoforms could increase our understanding of two distinct activation mechanisms. F1TnC was extensively characterized in the past, and its structure in solution was solved (19, 21). It was found to bind only one calcium atom in EF-hand 4, which undergoes a closed-to-open conformational change upon calcium binding. Only qualitative information was instead known previously for F2TnC.

This paper describes a comparison between F1 and F2TnC. We have now obtained full assignment of the backbone atoms both for full-length F2TnC and for the separate N and C lobes. The spectra of the fragments offer of course a better resolution. Given that the two chains are both EF-hand proteins and share a high level of sequence similarity, their different mechanical behavior cannot be explained by a different fold and must be linked to other properties, such as stability, dynamic behavior, or interactions. We provide a clear answer to account for all of these aspects.

F2TnC is overall more stable than F1TnC, having melting points of 40.6 °C and 74.6 °C for the apo and holo forms (25), respectively. Observation of single transitions suggests that the two lobes have similar stabilities. For comparison, the melting temperatures of apo F1TnC are 37.5 °C and 62.4 °C, respectively, whereas holo F1TnC undergoes an apparent single transition at a temperature of 56.5 °C. The difference in stability might have direct consequences on the mechanical properties of the two proteins.

An important difference between F1 and F2 TnC is their affinity for Ca^2+^. Both isoforms bind a single Ca^2+^ in EF-hand 4 in the C lobe, but the affinity of F1TnC for Ca^2+^ is 16 times that of F2TnC. The Ca^2+^ affinity of EF-hand 2 in the N lobe of F2TnC is low, and the ion is exchangeable under physiological conditions. This is consistent with the finding that the optimal Ca^2+^ concentration for stretch activation is *p*Ca 6, whereas maximal isometric force is produced at *p*Ca 4.5 (15). The Ca^2+^ affinities effectively separate the action of F1 and F2TnC in the muscle fiber. However, the optimal Ca^2+^concentration needed for stretch activation and development of isometric force is controversial. Other workers have found that *p*Ca 4.5 is needed for both stretch-activated and isometric contraction in *Lethocerus* (37) and *Drosophila* (38). The function of the C lobe in both isoforms is to anchor the N-terminal region of TnH. Although EF-hand 4 of F1TnC shows a small closed-to-open conformational change on binding Ca^2+^, binding of TnH(30–61) is not Ca^2+^-sensitive (22). A constant priming level of Ca^2+^ is necessary for stretch-activated contraction; removal of Ca^2+^ eliminates the response. However, it is not clear how F1TnC is turned off during the relaxation phase of oscillatory contractions, when the level of Ca^2+^ in the fiber remains constant. The N lobe of F1TnC is required for stretch activation, although it does not bind Ca^2+^ or TnH (19, 21).

The most striking difference between the two isoforms is in their ability to recognize TnH/TnI. The N lobe of F1TnC is not engaged in interactions with TnH/TnI, excluding the regulatory role for this domain observed in vertebrate TnC (19). The C lobe can bind both TnH(30–61) and TnH(126–159), which spans the sequence corresponding to the inhibitory and switch regions of vertebrate TnI, in a calcium-independent way (22). F2TnC has instead two distinct binding sites for TnH/TnI. It binds to TnH(30–61) with nanomolar affinity, which increases 5 times in the presence of Ca^2+^ (22). The N lobe can also bind this peptide but with lower affinity. TnH(126–159) binds preferentially the N lobe of F2TnC with micromolar affinity, which is 4 times greater in the presence of Ca^2+^ (25). Our results thus point toward an overall closer similarity between the complex of the F2 *Lethocerus* isoform and vertebrate cardiac and skeletal Tn, whereas F1TnC has distinct features. The differences between F1 and F2TnC could not have been anticipated on the basis of their sequences given the sequence similarity between them and, in general, all EF-hand proteins.

The information obtained from mapping our titrations onto the sequence of F2TnC allowed us to build a model for the hetero Tn trimer. This would be an easy task if the sequences could be aligned without major insertions/deletions, as is the case for TnC and TnH/TnI. TnT is quite different because the *Lethocerus* sequence is longer and the sequence alignment predicts an insertion in a region that would require a break in a helix. The insertion is too long to use standard comparative modeling methods (*e.g.* loop building). We thus used an iterative process that accommodates insertions in a gradual way without major perturbations of the overall structure. We were able to generate two different models of the complex based on the available high-resolution vertebrate structures. Although differing in the relative orientation of the TnC lobes, both models are stable throughout our MD sampling and compatible with all the information obtained, providing a starting point for future structural studies of the Tn complex. The resulting structural alignment (that is, the alignment based on the spatial correspondence of the chains rather than that obtained by maximizing the number of residue matches) is structurally reasonable as it places the insertion in the loop of the TnT hairpin, keeping the integrity of the secondary structure. As a result of this process, the relative angle between the two arms of the TnT hairpin changes slightly, and the head of the TnC dumbbell is forced to turn to avoid a clash with TnT.

Is this the correct structure of the F2-Tn complex in the sarcomere? We do not know at this stage, but, on the other hand, this is true also for the available crystal structures. The troponin complex is not made of rock-like globular proteins but of flexible chains that will be strongly influenced by the environment. Nevertheless, this and the crystal structures are excellent and indispensable starting points to use, for example, in validation-based fluorescence polarization studies (39, 40), which have been shown to be so successful for cardiac muscle.

In conclusion, our findings are clearly sufficient to explain how the role of the two complexes in muscle activation can be fine-tuned through their different properties and demonstrate again the incredible adaptability of the EF-hand fold to different functions. Our data also provide the rational basis for further studies that will finally allow us to understand the molecular mechanism of stretch activation.

## Experimental Procedures

### 

#### 

##### Sample Preparation

Full-length *Lethocerus* F2TnC and the two isolated N and C lobes, spanning residues 1–88 and 88–158, respectively, were produced by overexpression in *Escherichia coli*. The purification procedure was the same as the ones described previously (25). In short, all constructs were cloned in a pET24d (M11) expression vector (Novagen) containing an N-terminal hexahistidine tag followed by a tobacco etch virus protease cleavage site. The overexpressed proteins were purified by affinity using nickel-nitrilotriacetic acid resin (Qiagen) in two steps separated by an overnight tag cleavage step using in-house produced tobacco etch virus protease. The proteins were then passed through a size exclusion Superdex75 column in 20 mm Tris-HCl buffer at pH 6.8 with 100 mm KCl to remove any high molecular weight contaminants. Proteins were then concentrated on Vivaspin to 0.8 mm samples. Calcium depletion was achieved by a Chelex100 resin (Sigma) and adding 250 mm EDTA to the solution. The holo samples were obtained by adding a 5-fold molar excess of CaCl_2_.

##### Spectral Assignment

NMR spectra of F2TnC were acquired on ^15^N or ^15^N/^13^C uniformly labeled samples at 0.8 mm concentration in 90%/10% H_2_O/D_2_O and 20 mm Tris-HCl buffer (pH 6.8) and 100 mm KCl. All spectra were recorded at 25 °C on Bruker Avance II spectrometers operating at 600 or 800 MHz proton frequencies and equipped with 5-mm triple-resonance cryoprobes. ^15^N HSQC, HNCA, HN(CO)CA, HNCO, and HNCACB experiments were employed to obtain sequence specific ^1^H, ^15^N, ^13^Cα, ^13^Cβ, and ^13^C' full backbone assignments both on the full-length protein and the two fragments (41). Water was suppressed by the classical WATERGATE sequence (42). The spectra were processed and analyzed using NMRPipe (43) and NMRview (OneMoon Scientific). The ^1^H water signal was referenced at 4.76 ppm at 25 °C. The spectra were typically processed by applying a Gaussian window function and zero filled twice.

##### Relaxation Parameters

NOEs and longitudinal (T_1_) and transversal (T_2_) relaxation times were measured at 25 °C using a 0.8 mm sample on a Bruker Avance spectrometer operating at 600 MHz (22, 44). Eight (10, 100, 250, 500, 750, 1000, 1500, and 1800 ms) and eight (8, 16, 32, 48, 80, 128, 160, and 240 ms) spectra were recorded for the T_1_ and T_2_ measurements, respectively. T_1_ and T_2_ values were obtained by non-linear least squares fitting of the peak intensities to an exponential function. The analysis of Lipari and Zsabo (45) was carried out using Tensor2 software (46). Five possible models were tested iteratively for each amide proton. The data were fit with the order parameter S^2^ only (model 1); with S^2^ and the internal correlation time τi (model 2); with S^2^ and a chemical exchange Rex term (model 3); with S^2^, τ_I_, and R_ex_ (model 4); or with Sf2, Ss2, and τi (model 5), where the order parameter is split into two terms that correspond to a fast and a slow motion component. An initial estimate of the correlation time was obtained by removing all residues that contained a chemical exchange term. The estimated value was then carried out to fit every residue to the best model. Isotropic tumbling was assumed.

##### Peptide Titrations

^15^N-labeled samples of F2TnC were individually titrated with two synthetic peptides, TnH(30–61) and TnH(126–159). The concentrations of highly concentrated stock solutions of the peptides, in 100 mm KCl and 20 mm Tris-HCl, were measured in triplicate copies by amino acid analysis. The pH was adjusted to 6.8 with small additions of a 0.01 m NaOH solution. The pH was checked after the final peptide addition to make sure that it had remained constant throughout the titration. Complex formation was probed by recording ^1^H,^15^N HSQC spectra acquired at 600 MHz on an Advanced Bruker spectrometer equipped with a cryoprobe. The final molar ratios were 1:4 F2TnC:TnH peptide.

##### Structure Modeling

Structure modeling was carried out using the coordinates of the vertebrate skeletal and cardiac complexes as templates (PDB codes 1YTZ and 1J1D). We used a standard homology modeling protocol to build the TnC and TnH/TnI structures and an iterative gap growing process for TnT (47). Firstly, the *Lethocerus* sequence of chain T was built onto the template structure with the insertions missing. The trimer was then equilibrated in water for 20 ns before starting the gap growing process. Residues were then introduced at the insertions (2–5 at each time), allowing the system to relax after each addition with MD runs of 10–30 ns. Local secondary structure elements present at the inserted positions were conserved when introducing the new residues. Insertion 3 was modeled first, followed by insertion 2 and insertion 1.

Simulations of the two modeled trimers were carried out with the AMBER99SB-ILDN force field (48) solvated in Tip3p waters (49, 50). The system was equilibrated in water at 300 K with position restraints (force constant of 1000 kJ mol^−1^ nm^−2^) on the trimers for 10 ns and then simulated for 20 ns before starting the growing process. Every time new residues were inserted, the size of the box was readjusted and the system resolvated. Simulations were carried out with an integration step of 2 fs under periodic boundary conditions and LINCS constraints (51). Electrostatic interactions were calculated using the particle mesh Ewald method (52). All simulations were performed assuming constant number of particles, pressure, and temperature, coupling temperature with the V-rescale method (53) (coupling constant of 0.1 ps). Pressure coupling was carried out with the isotropic Berendsen method (54) (coupling constant of 1 ps, reference pressure of 1 bar). An additional simulation was carried out for 50 ns after the model was complete using a simulated annealing protocol to generate an ensemble of structures.

## Author Contributions

D. S. performed all NMR experiments and analyzed the spectra. M. S. H. and A. d. S. performed the modelling. B. B. provided the plasmids and the purification know-how. A. P. analyzed the data and wrote the article.

## Footnotes

2 The abbreviations used are:

Tm: tropomyosin
Tn: troponin
IFM: indirect flight muscle
MD: molecular dynamics
HSQC: heteronuclear single quantum coherence.
